# Reviewed article: Lopes LPN, Raimundo ACS, Caetano R, Lopes LC. Recalculation of the budgetary impact of risperidone use for Autism Spectrum Disorder in Brazil: a case study using measured demand data, 2017-2019. Epidemiol Serv Saude. 2025:34;e20240732.

**DOI:** 10.1590/S2237-96222025v34e20240732.b

**Published:** 2025-08-08

**Authors:** Osmar Cardoso

**Affiliations:** UFPI, Bioquímica e Farmacologia

**Table:** 

Rating	Excellent	Good	Average	Below average	Poor
Interest	✓				
Quality				✓	
Originality	✓				
Overall				✓	

**Table:** 

Questionnaire	Yes	No	Not applicable
Does the manuscript contain new and significant information to justify publication?	✓		
Does the Abstract (Summary) clearly and accurately describe the content of the article?		✓	
Is the problem significant and concisely stated?		✓	
Are the methods described comprehensively?		✓	
Are the interpretations and conclusions justified by the results?		✓	
Is adequate reference made to other work in the field?		✓	

## Manuscript Structure

Length of article is: Too short

Number of tables is: Adequate

Number of figures is: Too few

Please state any conflict(s) of interest that you have in relation to the review of this paper (state “none” if this is not applicable): None

## Comments

O trabalho apresentado é de uma temática muito importante e pouco estudada.

Mas alguns itens que devem ser melhor apresentados no trabalho,

Minhas preocupações com o artigo são:

1) O estudo não apresenta os links de acesso das fontes pesquisadas.

2) A utilização do termo resiliência na introdução que eu acho mais adequado a utilização da palavra resistência.

3) A figura 1 não é informativa.

4) Os dados permitem uma análise mais consistente com uma curva de tendência que poderia já pensar na população a ser atendida nos anos subsequentes.

5) Não foi apresentado um estudo de impacto, apenas um estudo de custos, porque não foi apresentado quanto esse aumento de custo impactou no orçamento do SUS e suas consequências.

6) Na apresentação do estudo foi colocado que era um estudo quali-quantitativo, mas não foi apresentado nenhum resultado qualitativo, o que também compromete a expectativa do trabalho.

Dessa forma, entendo que o trabalho deve ser repensado na sua forma estatística e de apresentação e deve ser novamente submetido quando estiver pronto. 

